# Environmental and genetic risk factors for MS: an integrated review

**DOI:** 10.1002/acn3.50862

**Published:** 2019-08-07

**Authors:** Emmanuelle Waubant, Robyn Lucas, Ellen Mowry, Jennifer Graves, Tomas Olsson, Lars Alfredsson, Annette Langer‐Gould

**Affiliations:** ^1^ Department of Neurology UC San Francisco San Francisco California; ^2^ National Centre for Epidemiology and Population Health, Research School of Population Health Australian National University Canberra Australia; ^3^ Department of Neurology and Epidemiology Johns Hopkins University Baltimore Maryland; ^4^ Department of Neurosciences UC San Diego San Diego California; ^5^ Department of Neurology Karolinska Institutet, Department of Clinical Neuroscience Stockholm Sweden; ^6^ Department of Epidemiology Institute of Environmental Medicine, Karolinska Institutet Stockholm Sweden; ^7^ Clinical & Translational Neuroscience Kaiser Permanente/Southern California Permanente Medical Group Los Angeles California

## Abstract

Recent findings have provided a molecular basis for the combined contributions of multifaceted risk factors for the onset of multiple sclerosis (MS). MS appears to start as a chronic dysregulation of immune homeostasis resulting from complex interactions between genetic predispositions, infectious exposures, and factors that lead to pro‐inflammatory states, including smoking, obesity, and low sun exposure. This is supported by the discovery of gene–environment (GxE) interactions and epigenetic alterations triggered by environmental exposures in individuals with particular genetic make‐ups. It is notable that several of these pro‐inflammatory factors have not emerged as strong prognostic indicators. Biological processes at play during the relapsing phase of the disease may result from initial inflammatory‐mediated injury, while risk factors for the later phase of MS, which is weighted toward neurodegeneration, are not yet well defined. This integrated review of current evidence guides recommendations for clinical practice and highlights research gaps.

## Introduction

Multiple sclerosis (MS) is a chronic inflammatory disease of the central nervous system (CNS). Whether this inflammation is in response to a chronic viral infection, primary neurodegenerative processes, or a reflection of a dysfunctional immune system was once hotly debated.^1^ In the past 5 years, consolidation of the evidence for environmental risk factors,^2^ identification of new genetic factors,^3^ and increased appreciation of the importance of gene–environment (GxE) interactions^4^ have led to a greater consensus. This new knowledge can provide clues to the causal biological pathways, and disease prevention for both populations and at‐risk individuals. The investigation of factors associated with disease progression and severity is similarly essential to guide the exploration of pathological pathways and, potentially, lifestyle recommendations to slow progression.

In this review, we discuss recent findings regarding genetic, epigenetic, and environmental risk factors for MS risk and prognosis, and how they impact our understanding of molecular processes at play. We also discuss new methods of analysis, proof‐of‐concept trials, and recommendations for at‐risk individuals and patients with the disease, as well as areas of future research needs.

## Literature Search Methods

We searched PubMed for papers published between 1 January 2013 and 1 April 2019, without any language restrictions, with the following terms: “environmental factors”, specific risk factors such as “EBV”, “vitamin D”, “sun exposure”, “genetic factors”, “gene‐environment interactions”, “epigenetics”, “multiple sclerosis”, “susceptibility”, “disease modification”, “modelling”. Additional references were gathered from retrieved publications, books, and conference reports. Original research articles and, when appropriate, high impact reviews were included. The final reference list was generated on the basis of relevance and originality with regard to the topics covered in this Review.

## Cause versus Course – Are the Risk Factors Different?

Genetic studies indicate the primary importance of the adaptive immune system in the pathogenesis of MS.^3^ Activation of microglia, probably as a result of a chronic inflammatory milieu, may in turn trigger neurotoxic pathways (e.g. production of reactive oxygen and nitrogen species) that drive neuronal damage and neurodegeneration.^5^ At all stages, inflammation and neurodegeneration probably coexist, with a weighting toward an inflammatory process in early MS, and toward neurodegeneration with disease progression.^6^ As such, risk factors for onset may differ from those for progression. There has been a wealth of research on risk factors for the onset of MS, but far less on risk factors for progression. This is at least partly because of the lack of long‐term, large cohort studies of people with MS that can provide prospective data on risk factors, to overcome the problem of reverse causality. In addition, it is possible that longer time elapsed between exposure to a risk factor and its effect on disease progression may further challenge such studies, as well as broad use of effective disease‐modifying therapies. For example, in a recent systematic review, some risk factors for onset, for example, low sun exposure/vitamin D, and smoking were also risk factors for relapse (low sun exposure/vitamin D), or for progression (smoking), but not for both.^7^ Others, for example, past infection with Epstein–Barr virus (EBV), were associated with onset but not clinical relapse or progression, while some, for example pregnancy, were associated with relapse, but not onset or progression. Here, for clarity, we discuss risk factors separately for onset, and for disease activity and progression.

### Risk factors for multiple sclerosis onset

#### Genes

That some people are genetically susceptible to developing MS and that the main susceptibility allele is a human leukocyte antigen (HLA) Class II gene have been known for over 50 years. Increasingly sophisticated genetic platforms have expanded our understanding by identifying over 200 non‐HLA single‐nucleotide polymorphisms (SNPs) and at least one protective HLA allele.^3^ Many of the non‐HLA SNPs are located near genes involved in adaptive or innate immunity underscoring that MS is a disorder of immune homeostasis. All of the SNPs so far identified are normal gene variants (i.e. not disease genes). Around 20% of the heritability risk is attributable to common genetic variants^3^ but low‐frequency and rare coding variation also contribute (about 5%).^3^

While several HLA Class II and I alleles contribute to MS susceptibility,^8^, ^9^ the main one is carriage of the *HLA DRB15:01* haplotype (odds ratio (OR) of ~3). This gene variant is carried by 25–30% of the population in northern Europe and the USA. The reasons for this association are still unclear. Hypothetically, the *DRB15:01*‐binding pocket may allow optimal binding and presentation of CNS‐related autoantigens, which drives T cells to attack the CNS. Autoproliferation of Th1 lymphocytes appears to be elevated in *HLA‐DR15*‐positive individuals and mediated by memory B cells in a HLA‐DR‐dependent manner.^10^ Interestingly, the *DRB15:01* gene is epigenetically regulated, leading to increased expression and antigen presentation.^8^

The second strongest MS gene variant is *HLA A02*, which is protective (OR ~0.6).^11^ The reasons for Class I‐associated protective effects are also unclear. The alleles of *HLA A02* may be important in the elimination of viruses connected to MS, such as EBV (see later). In addition, binding of a peptide from a protein commonly expressed by many cell types (glycerolphosphatidylcholine phosphodiesterase 1) to HLA‐A*02‐01 may cross‐stimulate a myelin‐reactive T‐cell receptor, possibly leading to increased CD8^+^ activity.^12^

For the many non‐HLA genetic loci associated with MS risk,^3^, ^13^ the magnitude of the association is much smaller (e.g. ORs up to 1.3) than for HLA. Furthermore, most of the SNPs are located outside exomes, suggesting that they, or nearby polymorphisms, are affecting the regulation of gene expression rather than the function of a gene product. Only a small number of non‐HLA influences in MS have so far been functionally deciphered: a genetically determined increase in the levels of soluble tumor necrosis factor (TNF) receptors blocks a protective action of TNF;^14^ a genetic polymorphism resulting in higher IL22 binding may neutralize a protective effect of IL22;^15^ and tyrosine kinase 2 exomic variation decreases pro‐inflammatory signaling.^16^ Many genetic variants are shared across several autoimmune diseases suggesting some common pathogenesis.^17^

Although most of the genetic work in MS has been conducted in populations with European ancestry, recent studies in African Americans have shown a significant overlap with MS variants reported in Whites.^18^

### Environmental factors (see also Table 1)

**Table 1:** Environmental factors associated with MS susceptibility and prognosis^a^

Risk factor	Susceptibility			Prognosis			
	Effect	Consistent across R/E?	Consistent across studies?	Disease activity		Disease progression	
				Relapses	New MRI lesions	Long‐term disability	Brain atrophy
Strong							
Prior EBV infection^33^	⇧	Yes	Yes	?	?	No	?
Cigarette smoking^33^	⇧	Yes	Yes	?	?	⇧	⇧/none
Moderate							
Low sun exposure^69^	⇧	Yes	Yes	⇧/none	?	?	?
Low 25OHD^33^	⇧/none	No	+/‐	No	⇧	?	No
High fish/PUFA intake^76^, ^77^, ^78^	⇩/none	Yes	+/‐	?	?	?	?
Pregnancy^29^, ^87^, ^91^, ^92^	⇩/none	Yes	No	⇩ preg/⇧ PP	⇩ preg/ ⇧ PP	No	?
Childhood/adolescent obesity^108^	⇧	Yes	Yes	?	?	?	Adolescent obesity
Weak							
Oral tobacco^98^	⇩	?	+/‐				?
Breastfeeding^29^, ^88^	⇩/none	No	No	No anyBF/ ⇩ exBF	?	?	?
CMV infection^33^	⇩/none	No	No	?	?	?	?
Major head injury^33^	⇧	?	No	?	?	?	?
Air pollution^93^, ^94^, ^95^, ^96^, ^97^	⇧/none	?	No	⇧/none	⇧/none	?	?
Organic solvents^116^	⇧	?	?	?	?	?	?

a Risk factors are grouped by strength and consistency of evidence of associations (or lack thereof) with MS susceptibility. Strong evidence: large meta‐analyses confirm the association, moderate evidence: several large studies suggest an association; weak evidence: some moderate size studies report an association. Evidence for association with prognosis is weak and mixed for most factors. ⇧, increased risk; ⇩, decreased risk, none: no effect on risk; ?, association unknown either due to the absence of evidence or sparse, very low quality evidence. MS, multiple sclerosis; R/E, race/ethnicity; EBV, Epstein–Barr Virus; CMV, Cytomegalovirus; preg, pregnancy; PP, postpartum period; anyBF, any breastfeeding; exBF, exclusive breastfeeding; 25OHD, serum 25‐hydroxyvitamin D levels; MRI, magnetic resonance imaging; dz, disease.

#### Life‐stage of risk of exposure

Based on migration studies,^19^ key environmental exposures associated with adult MS onset occur before age 15. New evidence suggests that risk exposures may occur in utero and in neonates.^20^, ^21^, ^22^, ^23^ For example, the risk of pediatric‐onset MS is increased in association with maternal illness other than diabetes and preeclampsia during pregnancy, and a father with an agricultural profession during pregnancy.^20^ Post‐winter birth is more common, and post‐summer birth less common, in people with MS,^24^ although there is some debate whether this finding may be due to residual confounding.^25^, ^26^ Lower vitamin D status during pregnancy or infancy is associated with increased risk of MS in Whites.^22^, ^23^, ^27^

Studies of cesarean section delivery and having been breastfed,^20^, ^28^, ^29^, ^30^, ^31^ both of which have profound effects on the microbiome, have yielded mixed results. Both deserve further study as these are modifiable factors. It is conceivable that some early life exposures may not contribute alone to MS onset, but are markers of risk that also act throughout childhood and teenage years.

#### Past viral infection and/or reactivation

The mechanisms through which past viral infections, particularly with herpes viruses, and viral reactivation may contribute to MS onset remain hypothetical.^32^

*Epstein–Barr virus*: there is strong, consistent, evidence across multiple racial/ethnic groups that past EBV infection, including infectious mononucleosis, positivity for EBV nuclear antigen (EBNA)‐1 IgG, or higher EBNA‐1 titers, is associated with an increased risk of MS.^33^ The high prevalence of EBV seropositivity in the general population suggests that any pathogenic effect of this agent may depend on other risk factors (e.g. GxE interactions). Recent work provides further evidence that EBV is causally involved in MS, possibly acting via EBNA‐2‐induced alterations in gene transcription in EBV‐infected astrocytes, microglia, and B cells (see Box ). An interaction between EBNA positivity and *HLA‐A02* has been reported.^34^, ^35^ In a recent study of MS cases and non‐MS controls who were seropositive for EBV, EBV viral load was lowest in HLA‐A*A02‐positive individuals, and highest in HLA‐B*07‐positive individuals. These findings support a causal role for EBV in MS, modulated by HLA‐Class 1 genotype through alterations in antigen presentation to T cells^36^. An additive interaction of EBV status with *DRB1* has also been reported to modulate MS risk in Whites^37^ and Blacks.^37^

Box 1 **EBV and MS ‐ what was known in 2013, and what is new in 2019**

**What was known?**

- EBV antibodies are present in almost 100% of people with adult MS.^38^
- Strong and consistent association between history or serological evidence (mainly anti‐EBNA IgG) of past EBV infection and increased risk of developing MS.^39^

**What is the view in 2019?**

- Meta‐analysis of meta‐analyses confirms consistent association of past history of infectious mononucleosis (OR = 2.17, 1.97–2.39) and anti‐EBNA IgG positivity (4.46, 3.26–6.09) and increased risk of MS.^33^
- In archived brain tissue, 93% of MS and 78% of control brains contained EBV latent membrane protein; 78% of chronic MS lesions contained EBV‐related proteins compared to 33% of non‐MS brains. About 85% of MS brains had frequent EBER‐positive cells; these were uncommon in non‐MS brains.^40^ Astrocytes and microglia as well as B cells were infected.^41^
- EBV encodes microRNAs (miRNAs) that are abundant in latently infected cells. EBV miRNAs inhibit the expression of viral antigens, allowing infected cells to evade the host's immune system. EBV miRNAs interfere with antigen presentation and immune cell activation thus suppressing the host antiviral immunity.
- Using novel computational methods, one study showed that EBNA2 protein occupies multiple loci as a transcription factor in MS, as well as a range of other autoimmune diseases including systemic lupus erythematosus, type 1 diabetes, inflammatory bowel diseases, rheumatoid arthritis, juvenile idiopathic arthritis, and celiac disease, suggesting genetic effects that are dependent on EBNA2.^42^ These findings are supported by recent experimental studies where by MS risk SNPs were overrepresented in the target loci of the EBV transcription factor EBNA2, in genes dysregulated between B and LCLs, and as targets for EBV miRNAs.

*Human Herpes virus 6 (HHV6)*: past infection with neurotropic HHV‐6 has been inconsistently linked with increased MS risk. A recent meta‐analysis of seven serological studies and 34 molecular studies that met high quality inclusion criteria showed an increased MS risk in association with evidence of past HHV‐6 infection.^43^

*Cytomegalovirus (CMV)*: A few studies have suggested a protective association of prior CMV infection with adult and pediatric MS while several have reported no association.^44^, ^45^, ^46^, ^47^

*Herpes simplex virus (HSV*): Children with prior exposure to HSV‐1 have a modestly increased risk of having pediatric MS. This is mostly seen in Whites and *DRB1*‐negative individuals.^44^

*MS associated human endogenous retroviruses (HERVs)*: Expression of HERVs is curtailed in healthy individuals by epigenetic factors, but HERVs are transcribed at high levels in autoimmune diseases such as rheumatoid arthritis, Sjogren's disease, systemic lupus erythematosus, and MS.^48^ HERVs are expressed in the CNS; some seem particularly associated with MS susceptibility, others with disease course.^48^

#### Sun exposure and vitamin D

That MS prevalence increases with greater distance from the equator led to the hypothesis that lower exposure to ultraviolet (UV) radiation and subsequently, lower vitamin D status (blood concentration of 25‐hydroxyvitamin D (25OHD)), increases the risk of MS. Latitude gradients have reduced over time in some populations,^49^, ^50^ but not all;^49^, ^51^ this may be due to changes in exposure to latitude‐related factors, for example, sun exposure, diet, in some populations, but not others. Higher latitude^52^ and lower summer sun exposure in adolescence^53^ have been linked to a younger age of onset.

Observational studies suggest higher MS risk with lower sun exposure in childhood,^54^, ^55^, ^56^, ^57^ adolescence,^54^, ^58^ adulthood,^28^, ^59^, ^60^ and over the whole life.^59^, ^61^, ^62^, ^63^ The findings are relatively, but not completely,^64^ consistent, despite the wide range of ways of measuring sun exposure.

Observational studies similarly show that lower 25OHD levels are associated with increased MS risk in White populations.^22^, ^59^, ^65^, ^66^, ^67^ A similar association is not seen in Hispanics and Blacks.^68^ Within White populations, the dose–response relationship is inconsistent; for example, both threshold (e.g. protective only at 25OHD levels >99 nmol/L^65^ or increased risk only for the lowest quintile of 25OHD^69^) and linear effects are described.^66^ These inconsistent associations may be because few studies have accounted for sun exposure. Where data are available on both sun exposure and 25OHD levels, there are statistically independent benefits of higher levels of either in Whites.^59^, ^68^ Both UV radiation and vitamin D have effects on innate and adaptive immune function that would plausibly be beneficial for MS.^70^

It can be difficult in observational studies to distinguish between the effects of low sun exposure and low vitamin D. Sun exposure may be a marker of lifetime vitamin D status; equally, 25OHD level may simply be an objective marker of sun exposure. Vitamin D supplementation leads to a rise in 25OHD levels without a co‐intervention of sun exposure; thus, supplementation studies can evaluate the specific impact of vitamin D on relevant outcomes. As an illustration, observational data from the Finnish Maternity Cohort showed a 50nmol/L increase in 25OHD was associated with a 39% lower MS risk, with blood samples taken on average ~9 years prior to MS onset.^67^ Routine vitamin D supplementation for pregnant women was introduced in 2004, providing a natural trial of vitamin D supplementation and the impact on MS incidence. Incidence rates for MS in southwest Finland were 5.1, 5.2, and 11.6 per 100 000 for the hospital districts of Uusimaa, Vaasa, and Seinajoki, respectively, from 1979 to 1993,^71^ and 12.1 per 100 000 for southwest Finland in 2012–2016.^72^ This is not the dramatic reduction in MS incidence that might have been expected with the advent of vitamin D supplementation. However, supplementation may have been relatively low (Nordic recommendations are for 400 IU/day).

##### Diet and supplements

In vitro and animal studies suggest that lower intracellular sodium concentrations and sodium intake may have protective effects on immune function.^73^ However, recent case‐control studies show no association between dietary sodium intake and risk of MS.^74^, ^75^

Polyunsaturated fatty acids (PUFAs) are strong immunomodulators in vitro and in animal models. Consistent with this, most, but not all,^76^ studies that measured seafood‐based sources or direct micronutrient PUFA intake found a reduced risk of MS with higher fatty fish intake,^77^, ^78^ or cod liver oil supplementation.^79^ These findings should be interpreted cautiously, with inconsistencies across studies in the timing of the risk exposure, that is, during childhood, adolescence, or through adulthood. In addition, most studies could not fully account for a substitution effect (i.e. it may not be fish that is protective but what is not being eaten instead of fish that is harmful).

##### Gut microbiota

Dysbiosis in the gut microbiota has emerged as a potential risk factor for MS, following the report of its key role in shaping the immune response. Many factors modulate the gut microbiota including diet, obesity, antibiotic use, and cigarette smoking. Studies of modest size have reported that gut microbiota diversity is overall similar in prevalent MS cases and controls, including between monozygotic twin pairs discordant for MS.^80^, ^81^, ^82^, ^83^, ^84^, ^85^, ^86^ Discrete taxonomic enrichments and depletions have been observed in MS cases compared to controls, some of which vary across studies. The discrepancies across studies could be due to differences in inclusion criteria and disease duration, as well as in the analytic platforms used. In general, the reported discrete taxonomic differences point toward a more pro‐inflammatory milieu in cases. Whether this is a potential cause, or consequence of MS, treatments, or dietary changes following diagnosis, has not been determined.

##### Reproductive factors

Sex dimorphism is predominant between puberty and menopause. Before and after these reproductive milestones the sex ratio is 1:1; between them the ratio is 3:1. Earlier age at menarche is associated with an increased risk of MS,^29^, ^87^ and the onset of pediatric MS peaks 2 years after menarche.^88^ Whether this is mediated by complex interactions between immunological responses to infections (e.g. EBV), direct effects of sex or other hormones, or other factors is unknown. The effect of menopause on disease course and MS susceptibility is unclear and worthy of further study. One cross‐sectional referral center study suggested a decrease in female:male ratio in patients with symptom onset after age 50.^89^ Whether this is related to a decline in risk of relapsing‐onset MS in postmenopausal women or an increase in risk of progressive‐onset MS in older men is unclear. Of note, recently updated MS prevalence estimates in the USA show only a slight decrease in female:male ratio over age 55.^90^

Two recent studies suggest that women who breastfeed their infants exclusively^87^ and for a prolonged period^29^ may have a lower subsequent risk of MS. How breastfeeding could protect the mother against an autoimmune disease is unknown. The potentially protective effect of breastfeeding may explain the inconsistent association between parity and MS risk, either protective^87^, ^91^ or no association,^29^, ^92^ as most studies did not account for subsequent breastfeeding.

Other behavioral reproductive factors including use of hormonal contraceptives (when smoking is adequately accounted for), age at first birth, and total ovulatory and menstrual years do not appear to be associated with MS risk.^91^

##### Air pollution

Whether air pollution increases the risk of MS is unclear. Exposure to particulate matter (PM) triggers an inflammatory response in the lung, with the release of inflammatory cytokines and elevated systemic levels. Long‐term exposure to air pollution is associated with neuroinflammation and damage to the blood–brain barrier. Studies examining exposure to PM of aerodynamic diameter <10 µm (PM_10_) show an increased risk of MS or no association.^93^, ^94^, ^95^ A strong association between air pollution (but not water or land pollution) and having pediatric MS^96^ has been reported and attributed to exposure to sulphur dioxide, PM_2.5_, carbon monoxide, and lead.^97^

##### Smoking and oral tobacco

Cigarette smoking is a well‐established risk factor for MS onset, with a clear dose–response relationship.^98^, ^99^ Age at starting smoking does not seem to affect MS risk.^98^ However, quitting smoking is associated with a gradual decline in the excess risk of MS, to zero at 10 years post‐cessation, regardless of the cumulative dose.^98^ Passive exposure to smoking, including water pipe smoking, has also been associated with increased MS risk.^100^, ^101^ In contrast, snuff, a form of smokeless tobacco, has been associated with a dose‐dependent lower MS risk, particularly among smokers.^98^ This suggests that exposure to tobacco smoke is due to nonspecific lung irritation and inflammation. Nicotine itself may have neuroprotective effects as reported in experimental models.^102^ The harmful effects of tobacco smoke may be conveyed through peribronchial lymphatic tissue in licensing autoreactive T cells or activation of the aryl hydrocarbon receptor, as is suggested in rheumatoid arthritis.^103^

##### Childhood/adolescent obesity

Several high quality observational studies have reported that obesity in adolescence and early adulthood is associated with approximately double the risk of developing pediatric and adult MS compared with normal‐weight individuals.^104^, ^105^, ^106^ The association has been largely confirmed in females, including a dose‐effect,^105^ while evidence is mixed regarding the association in males.^106^, ^107^, ^108^, ^109^

##### Mendelian randomization (MR) studies

Three MR studies have shown that genetically lower 25OHD levels are significantly associated with increased MS risk albeit only in White, non‐Hispanics.^104^, ^110^, ^111^ Critical MR assumptions were carefully considered.^112^, ^113^ As confounding by ancestry could occur,^114^ the vitamin D MR models were rigorously adjusted for population stratification and sensitivity analyses were performed to assess ancestry's potential impact, since several of the vitamin D‐related variants have different frequencies in different ethnic groups, and variation in risk of MS according to ethnicity is well‐described. Genetic risk scores of variants associated with obesity in large (gene wide association study) GWAS studies have confirmed a strong association with pediatric and adult MS, suggesting causality.^104^, ^115^

#### GxE interactions and MS onset

Environment interactions with genotype in MS have focused on HLA DRB1*15:01. Table 2 summarizes current knowledge of interactions between environmental factors and carrying HLA DRB1*15:01 and lack of HLA A*02. Thus far, interactions between *HLA DRB1* and EBV and HSV infections, smoking and adolescent obesity, but not oral nicotine or CMV, have been demonstrated in Whites. The interaction between EBV serology and *HLA DRB1* was recently confirmed in Blacks.^45^ Occupational exposure to inhaled organic solvents may also interact with both HLA DRB1*15:01 and lack of HLA A*02.^116^ If these findings are confirmed, it would indicate that the general lung irritation could contribute to MS risk.

**Table 2:** Interactions between the main MS susceptibility genes and environmental factors associated with MS risk in White, non‐Hispanics (adapted from^181^)

Factor	Odds ratio (95% CI)	Source of estimate	Interaction with HLA‐DR15 genes	Combined OR (nongenetic factor + HLA)	Source of estimate	Immune system implicated
EBV serology (+ vs. −)	4.5 (3.3–6.6)	Meta‐analysis^182^	Yes	6.1 (3.8–9.7)	Meta‐analysis^37^	Yes
Infectious mononucleosis (yes vs. no)	2.2 (2.0–2.4)	Meta‐analysis^183^	Yes	7.0 (3.3–15.4)	Single study^184^	Yes
Smoking (ever vs. never)	1.5 (1.3–1.6)	Meta‐analysis^99^	Yes	7.4 (6.7–8.3)	Pooled analysis^117^	Yes
Passive smoking (among nonsmokers; yes vs. no)	1.1 (0.9–1.4)	Meta‐analysis^99^	Yes	4.7 (3.2–5.8)	Single study^101^	Yes
Organic solvent exposure (ever vs. never)	1.5 (1.0–2.3)	Meta‐analysis^185^	Yes	6.7 (3.7–12.1)	Single study^116^	Yes
Oral tobacco/nicotine (ever vs. never)	0.83 (0.75–0.92)	Two case‐control studies^186^	No	NA		Yes
Adolescent BMI (at 20 years, ≥30 vs. 18.5‐<21)	2.1 (1.5–3.0)	Multiple studies^187^	Yes	16.2 (7.5–35.2)	Single study^188^	Yes

ORs are from meta‐analysis where that is available; we cite the latest papers published, and by preference those using incident cases (i.e. newly diagnosed). BMI, body mass index; EBV, Epstein–Barr virus; HLA, human leukocyte antigen; MS, multiple sclerosis; OR, odds ratio.

While our genotype cannot be altered, smoking is a nice example of how MS risk can be compounded or reduced even in those with a genetic susceptibility for MS. Nonsmokers who carry HLA DRB1*15:01 and lack HLA A*02 have a combined OR of ~5; however, among smokers, the combined OR is ~14, much higher than the sum of the main effects associated with each factor.^117^ Smoking may also interact with the *NAT1* gene (encoding N‐acetyltransferase 1 enzyme that is important in the metabolism of aromatic amines present in cigarette smoke) to alter MS risk.^118^

These findings support the notion that MS onset requires the interaction between multiple pro‐inflammatory states and help explain why MS is rare, but carrying HLA DRB1*15:01 and pro‐inflammatory exposures, like EBV infection, smoking, and obesity, are common.

#### Epigenetics and MS onset

Epigenetics refers to heritable changes outside of the DNA sequence that influence which genes are turned on and off and when. Epigenetic changes reflect aging and our body's interaction with the environment. In some instances, these changes can contribute to specific diseases. Little is known about how such changes influence MS susceptibility or prognosis. DNA methylation is probably the best explored of the three main epigenetic processes, and aberrant methylation in gene regulator regions may underlie processes involved in MS onset.^119^ While methylation changes have been reported in MS at *HLA‐DRB1* in CD4_+_ but not CD8^+^ T cells,^120^, ^121^ in brains,^122^ and in relapsing‐remitting versus primary progressive MS,^123^ it is unclear if these changes are drivers or responses to the disease.

Expression of several microRNAs (miRNAs) appears to be increased in various tissues from patients with MS and may alter the immune response and ultimately contribute to disease onset.^124^, ^125^

Increasing our understanding of epigenetic changes, particularly reversible ones, may elucidate pathogenic pathways and guide the development of new therapeutic strategies.

### Risk factors for disease progression

Identifying clear‐cut MS prognostic factors is challenging because disability accumulates slowly over decades and there is a lack of good surrogates of disability progression. Disease activity (rate of relapses and new MRI lesions) is thought to reflect inflammatory processes, whereas disease progression (worsening of disability and brain and cord atrophy) may better reflect neuronal injury.^6^ An additional challenge is that large population‐based MS cohorts that represent the broad range of MS prognosis are rarely associated with genetic and environmental exposure data. Most studies discussed herein are highly selected, referral center populations and utilize anatomical correlates of inflammation (T2 and contrast‐enhancing lesions) and neurodegeneration (cerebral atrophy) or relapse rates. Treatment decisions are often based on relapse rate or the development of new MRI lesions. However, while relapses in the first 5 years of disease onset are associated with short‐term disability progression, they contribute to a lesser extent to long‐term disability progression.^126^, ^127^ While spinal cord, and to a lesser extent global brain, atrophy measures are correlated with disability progression, they are not yet broadly implemented for treatment decisions.^126^ Finally, cord atrophy, although a very promising predictor of disability progression, has not yet been used as a prognostic marker in studies of the effect of genetic or environmental factors, as its development is more recent than brain atrophy.

#### Genes

There have not been consistent associations of genetic susceptibility factors with cross‐sectional disability metrics or standard MRI outcomes. In a study of over 7000 individuals with MS, a genetic burden score was not associated with disability.^128^ Other smaller studies have reported associations of several genetic variants and progression of disability but have not yet been replicated.^129^, ^130^, ^131^, ^132^. Notably, no study has been able to identify consistently a significant difference in genetic burden or variants between relapsing and progressive‐onset MS.^11^, ^133^

Though overall susceptibility genes do not appear to have large effects on phenotype, there is some evidence that HLA and non‐HLA genetic risk factors may alter relapse rates, although overall findings are mixed and weak. Some susceptibility polymorphisms have been associated with attack or MRI lesion location and severity.^134^, ^135^, ^136^, ^137^ The strongest genetic factor (HLA‐DRB1*15:01) has been associated with earlier age of onset and possibly with greater deep gray matter atrophy.^138^ The MS susceptibility polymorphism within the Abelson Helper Integration site 1 gene (*AHI1*) has been associated with higher relapse rate in two separate cohorts (one of children and another of adults), with similar effect size.^139^ In a genome wide search for alleles associated with relapse rate, a SNP within the gene *LRP2* was associated with a twofold increase in the hazard to relapse in three independent MS cohorts, including adults and children.^140^ *LRP2* is located on the cell surface of neurons and oligodendrocytes and participates in axon guidance. In early MS, a genetic variant within myelin basic protein (MBP) was reported to be associated with both relapse hazard and disability progression.^129^ Genetic factors within the vitamin D pathway have been associated with both 25OHD levels and relapse rate.^141^, ^142^

#### Environment

##### Sun exposure and vitamin D

Few studies have tested the association between sun exposure and relapse rate and/or progression, while it has been hard to rule out reverse causality in the association of lower 25OHD with more active disease course.

In a population‐based prospective cohort study, higher sun exposure prior to MS onset and/or increasing sun exposure after the first MS relapse (onset attack) were associated with a longer first‐to‐second attack interval and lower relapse rate.^143^ Ecological studies show higher relapse rates during seasonal low ambient UV radiation,^144^ and a stronger seasonal pattern (peak in early spring) at higher latitudes.^145^

A recent comprehensive review of vitamin D and MS concludes that a low serum 25OHD level is associated with increased disease activity in MS, but the findings are typically stronger for MRI than clinical outcomes.^66^ Most (but not all) observational studies show lower relapse rates and (Expanded Disability Status Scale) EDSS in association with higher 25OHD level, although this may be disease‐induced rather than causal.^66^

Randomized controlled trials (RCTs) of vitamin D supplementation have not shown the hoped‐for benefits. A recent meta‐analysis of RCTs of vitamin D supplementation in people with MS suggests no therapeutic effect on either EDSS score or annualized relapse rate.^146^ Indeed, a previous meta‐analysis suggested that higher doses of vitamin D supplementation may be associated with worse outcomes.^147^ It is important to note that many of the RCTs of vitamin D supplementation for people with MS have had limitations of small sample size (low power), short duration, and including people who are not vitamin D deficient. Studies in other diseases have shown that benefits of vitamin D supplementation are seen only in those who are severely deficient at baseline.^148^ Additional studies of vitamin D supplementation in relapsing‐remitting MS and CIS are currently underway.

The possible benefits of ultra violet B phototherapy have recently been tested in a RCT of people with clinically isolated syndrome. Following an 8‐week course of phototherapy, there was a 30% reduction in risk of converting to MS at the 12‐month follow‐up. This finding is promising, although it was not statistically significant, likely due to a small sample.^149^

##### Diet

Despite great interest, there are few studies of dietary effects on MS prognosis, all with significant limitations. The potential pro‐inflammatory effects of a higher salt intake observed in animal models have yielded mixed results in humans.^73^ Higher sodium levels in random urine samples were associated with higher rate of new MRI lesions and relapses,^150^ yet 24‐hour urine sodium levels, and salt intake from food frequency questionnaires showed no association.^151^, ^152^ In pediatric MS, a higher saturated fat intake and lower vegetable intake were independently associated with higher relapse risk.^153^

Small diet intervention trials have not shown any effect of low‐fat diet on relapse rate or MRI activity, but have suggested a possible benefit on fatigue.^154^ Trials of omega‐3 supplementation have not shown any effect on disease activity.^155^ Randomization to calorie restriction diets was associated with improvement of emotional well‐being compared to controls, but the study was too short to evaluate risk of relapse.^156^

#### Pregnancy, breastfeeding, and other reproductive factors

There is strong evidence that pregnancy does not worsen long‐term prognosis,^157^, ^158^, ^159^ despite an increased risk of relapses in the early postpartum period.^160^ It is unclear if pregnancy improves MS outcome. Exclusive breastfeeding (no supplemental feedings for at least 2 months) reduces this risk of postpartum relapses, whereas nonexclusive breastfeeding appears to have no effect.^161^ Whether breastfeeding influences long‐term MS prognosis is unknown.

It is unclear whether hormonal contraceptive use or age at menarche affect long‐term prognosis as the sparse data inadequately controlled for other factors. In girls with MS, there may be a small increase in the risk of relapse during the peri‐menarche year.^88^

##### Air pollution

A few studies have shown an association between PM_10_ levels and risk of MS relapse or MRI activity.^162^, ^163^, ^164^, ^165^ A deeper look at various pollutants suggested an increased risk of MS relapse following recent exposure to nitrogen dioxide (particularly during the cold season) or ozone (particularly for the hot season).^164^ Benzene and carbon monoxide were not associated with MS relapses. Finally, worse air pollution in Iran was associated with poorer recovery from a first MS event.^166^

##### Obesity

Whether obesity is associated with a higher risk of MS‐related disability is unclear. Obesity is a low‐grade inflammatory state^167^ and could act as a pro‐inflammatory cofactor resulting in an earlier age at onset and more aggressive inflammation. However, the evidence is sparse, mixed, and weak. Obesity prior to disease onset is associated with younger age at onset only in women^168^ and earlier age at conversion to secondary progressive MS only in smokers,^169^ both with marginal effects and inadequate statistical analyses. Studies in highly selected MS populations found that higher body mass index (BMI) at diagnosis or baseline has either no effect^170^ or harmful effects^171^ on brain atrophy, yet current BMI is not associated with the current level of disability.^172^ In addition to obesity, several studies have focused on the association of comorbidities with MS progression but these will not be reviewed.

### Prospects for prevention, prognostication, and intervention

Although the risk of MS is higher in patient's first‐degree relatives, most first‐degree relatives will never develop the disease. There is currently no genetic testing that is recommended for at‐risk individuals as most genetic variants associated with MS are normal genes and most carriers will remain MS free.

The following recommendations are not based on definitive proof‐of‐concept intervention trials as these are difficult to implement in large cohorts, but are derived from epidemiological findings with the caveat that these studies do not provide precise duration and dose of exposures that may have preventative benefits. Table 1 highlights the gaps in the evidence and the room for future research. Exposure to cigarette smoking, low sun exposure, and childhood obesity are all modifiable risk factors that have been repeatedly and consistently associated with an increased risk of MS. Diets high in fish (and low in saturated fats) are recommended as part of a heart‐healthy diet and may also reduce the risk of MS. Thus, recommendations for at‐risk individuals are consistent with those for general health benefits and may reduce the risk of MS: avoidance of exposure to first‐ and second‐hand cigarette smoke; using the UV index to guide sun exposure in accordance with WHO guidelines,^173^ but ensuring regular sun exposure below a sunburning dose on most days of the week;^174^ and preventing childhood obesity by promoting a Mediterranean diet and exercise. Many clinicians also recommend vitamin D supplementation to prevent MS, although the hopes of general health benefits are probably limited aside from preventing rickets or osteoporosis in those individuals with extremely low levels (e.g. <25 nmol/L). While vitamin D_3_ supplementation doses of up to 4000 IU/day (as commonly used in MS) do not appear to be harmful,^175^, ^176^ this should not be viewed as a replacement for sun exposure, particularly in non‐Whites, as sun exposure may provide additional benefits (see Sun exposure and Vitamin D section).

For patients with established MS, some modifications of lifestyle factors can be recommended with reasonable confidence for their likely benefit to overall health, even if MS‐specific benefits are unclear. These include smoking cessation, achieving and maintaining normal body weight, and osteoporosis prevention.

Obesity or obesity‐related comorbidities have been associated with worse outcomes among people with MS.^177^ The health benefits of a Mediterranean‐style diet for such comorbidities are well‐established.^178^ Thus it is reasonable, in the absence of a specific “MS diet” that is shown in well‐designed trials to reduce the risk of relapses or disability, to advise patients with MS to adopt a Mediterranean‐style diet as a means of reducing obesity‐related comorbidity risk.^179^

There is no evidence that pregnancy detrimentally affects long‐term MS disability; thus, women with MS who want to have children should be reassured and supported through this life phase. Most women with newborns should be encouraged to breastfeed if they desire. Breastfeeding exclusively may reduce the risk of postpartum MS relapses; however, certain MS treatments should not be resumed while breastfeeding.^180^

## Conclusion

We have come a long way over the past 50 years in the understanding of risk factors for MS onset and progression. These appear to be extremely complex, and individually heterogeneous. Many genetic variants, most with modest effects, have been reported, but their precise biological role remains to be clarified. Similarly, it is likely that many environmental risk factors are also at play, possibly with modest effects as well. Causality remains challenging to establish for many environmental factors. Trans‐ethnic and newer analysis strategies have helped strengthen evidence for EBV, obesity, and sun exposure, and yet point to low vitamin D as a pro‐inflammatory cofactor only in Whites. While it is conceivable that some environmental factors may have several biological downstream effects relevant to disease processes (i.e. air pollution could decrease sunlight exposure and activate biological pathways such as the oxidative and aryl hydrocarbon receptor pathways), it is also expected that various environmental exposures could act on a limited number of common efferent pathways (i.e. result in similar biological effects). Nevertheless, despite recent progress, these efferent pathways remain unclear, both for susceptibility (onset) and disease progression. RCTs currently underway, and innovative designs, for example, for dietary interventions, and analysis/modeling, for example, mediation analysis for modeling epigenetic changes as a result of environmental influences, should help to clarify these pathways and beneficial causal influences.

## Authors Contribution

All the authors have performed literature search and review, drafted various sections of the first draft, including tables and figure. The authors have provided edits for the entire manuscript.

## Conflict of Interests

E. Waubant has not received any pharmaceutical company honorarium. She is site PI for a Novartis and Roche trial. She has volunteered on an advisory board for a Novartis trial. She is a non‐remunerated advisor for clinical trial design to Novartis, Biogen‐IDEC, Sanofi, Genentech, Serono and Celgene. She has funding from the NIH, NMSS, PCORI, and the Race to Erase MS. She is the section editor for Annals of Clinical and Translational Neurology, and co‐Chief editor for MSARD. R.M. Lucas has not received any pharmaceutical company honoraria. She was an invited speaker at CMSC 2018, with travel and accommodation paid. She received funding from MS Research Australia and the National Health and Medical Research Council of Australia. Her salary is part‐funded through a National Health and Medical Research Council of Australia Senior Research Fellowship. E. Mowry receives research support from Biogen and Genzyme. She was site PI for clinical studies sponsored by Biogen and Sun Pharma. Teva Neuroscience provides free medication for a clinical trial, of which she is PI. Dr. Mowry receives honoraria for editorial duties for UpToDate. J. Graves has received honoraria for non‐promotional, unbranded educational seminar speaking for Biogen and Genzyme. She has research support from Biogen and Genentech. T. Olsson has received unrestricted MS research grants, and/or compensation for lectures and advisory boards from Biogen, Novartis, Genzyme, Roche and Merck. L. Alfredsson receives research support from the Swedish Research Council and the Swedish Research Council for Health, Working life and Welfare. He has received speakers honoraria from Teva and Biogen. A. Langer‐Gould reports grants from National Institutes of Health, grants from Patient Centered Outcomes Research Institute, grants from National MS Society, non‐financial support from European Congress for Treatment and Research in MS (ECTRIMS), non‐financial support from Institute for Clinical and Economic Review, other from Biogen, from Roche, outside the submitted work.

## Panel 1: Definition of Terms

**Causality**: the attribution of change in risk of the outcome to a specific exposure, that is, there is sufficient evidence to say that having the exposure changes the (future) risk of getting the outcome of interest.

**CNS‐related autoantigens**: include myelin basic protein (MBP), proteolipid protein (PLP), and myelin oligodendrocyte glycoprotein (MOG).

**Confidence interval**: 95% confidence interval (CI): the OR (or any effect estimate) is a “best estimate” of the true value of the parameter (here the OR). The 95% CI tells us that “the frequency with which the interval will contain the true parameter will be at least 95%”^189^ For an OR, if the 95% CI contains 1.00, the finding is not statistically significant at *P* < 0.05.

**Confounder**: a factor that is statistically associated with the exposure of interest and is a cause of the outcome of interest, but does not lie on the causal pathway from the exposure to the outcome. Failure to adjust for confounders may lead to an incorrect estimate of the effect of the exposure on the outcome.

**Disease modifiers**: Genetic and environmental factors that change the course of MS (relapse rate, rate of new lesions on brain MRI, progression of disability, progression of brain atrophy).

**Epigenetic changes**: Epigenetic changes, including DNA methylation, histone modification, and microRNA‐associated posttranscriptional gene silencing, alter gene expression without altering the DNA sequence. Epigenetic mechanisms play a key role in mediating the effects of a range of environmental exposures, for example, the effects of smoking on human health.

**Gene–environment interactions**: When assessing interactions between causal factors, departure from additivity of effects is used as a criterion (Rothman 1980, 2008), which, if present, indicates action of the two factors on the same biological pathway. In simple terms, interaction is present when the risk of developing disease among those exposed to both (causal) factors is higher than the risk expected on the basis of the sum of the absolute effects attributed to each factor individually.

**HLA genes**: The HLA gene region on human chromosome 6 contains around 200 genes, the majority contributing to immune function. The main actors in most immune‐mediated diseases are the Class II and I genes which contribute to the presentation of antigenic peptides to T cells, Class II to CD4^+^ T cells, Class I to CD8^+^ T cells. Activation of T cells following recognition of the peptides can be beneficial, for example, against infection or tumors, or adverse, if the peptides are self‐peptides, potentially leading to autoimmunity. HLA Class II and I genes are the most polymorphic genes suggesting an evolutionary pressure to maintain differences within the species to escape infections. The differences in the genetic code for different alleles depend on nucleotides resulting in different binding abilities in the antigen‐binding pocket. There are several Class II genes: *HLA DRB*, *HLA DQ,* and *HLA DP*. Class I molecules are instrumental in the immune defense against infections, killing virus infected cells. However, they can also have suppressive functions, for example, certain alleles of Class I molecules in rat experimental autoimmune encephalomyelitis (EAE) confer protection through CD8^+^ cells producing transforming growth factor beta (TGFβ) Each immune‐mediated disease has its own HLA association pattern.

**Mendelian randomization**: Mendelian randomization (MR) is a method of using data on genetic polymorphisms that lead to variations in levels of a risk factor as an indirect measure of lifelong exposure. Thus, several GWAS have described polymorphisms in vitamin D metabolism genes that account for higher 25OHD levels (e.g.^190^, ^191^). A requirement of MR studies is that the instrumental variable (i.e. the genetic polymorphisms) can only affect the outcome by altering the exposure they are an instrument for.^192^ That is, the genes involved in the genetic “score” for higher 25OHD levels that is used as an instrumental variable should not affect the risk of MS through any other pathway than causing higher 25OHD levels.

**Odds ratio**: the odds ratio is an estimate of the increased or decreased risk of disease. A value of >1.0 indicates an increased risk of having the disease; an OR <1 indicates a reduced odds of having the disease (i.e. a protective effect). The value of the OR can be quantitatively interpreted as the percentage increase/decrease in the odds of having the disease for a one‐unit increase in the exposure, for example, an OR of 1.20 for ever smoking versus never smoking can be interpreted as a 20% increase in the odds of disease comparing those two categories.

**Susceptibility factors**: Genetic and environmental factors that alter the risk of developing a disease.

**UV irradiance**: levels of ambient UV radiation decrease with increasing distance from the Equator.

**Vitamin D status**: Vitamin D status is measured by the blood concentration of an intermediate metabolite, 25‐hydroxyvitamin D (25OHD). Because the 25OHD concentration depends on the ambient UV radiation as well as time in the sun and how much skin is exposed, there may not be a clear latitude gradient (where measurements use a standardized protocol for sample collection and assay). Importantly, the 25OHD concentration is a marker of both vitamin D status and of recent sun exposure, and questionnaire measures of sun exposure are used as a proxy for past vitamin D status.
